# Rhabdomyolysis in Acute HIV Infection

**DOI:** 10.7759/cureus.64393

**Published:** 2024-07-12

**Authors:** Yemesrach F Mekonen, Maria V Perez, Maria C Tole, Osmaickel Redondo, Mahmoud Ali

**Affiliations:** 1 Internal Medicine, St. Barnabas Hospital (SBH) Health System, Bronx, USA

**Keywords:** myalgia, highly active antiretroviral treatment, creatine phosphokinase, acute hiv infection, rhabdomyolysis

## Abstract

Rhabdomyolysis is a rare but potentially life-threatening complication of acute HIV infection. We present a case report of a young adult male who presented with fever, myalgia, and elevated creatine phosphokinase levels, ultimately diagnosed with acute HIV infection-associated rhabdomyolysis. This case highlights the importance of considering HIV infection in the differential diagnosis of rhabdomyolysis, particularly in at-risk populations, even in the absence of typical HIV-related symptoms.

## Introduction

Rhabdomyolysis is defined by the disintegration of skeletal muscle fibers and the subsequent leakage of muscle cell contents into the circulation [1]. The mechanisms behind skeletal muscle injury can be categorized into hypoxic, physical, chemical, or biological insults, with the most common causes being prolonged exertion during exercise, trauma, or alcohol abuse [1]. Since skeletal muscle makes up forty percent of the total body mass, significant injury may cause an extracellular buildup of cellular content that overwhelms the body's clearance processes. This can lead to myoglobinuria, electrolyte abnormalities, and often acute kidney injury (AKI), which develops into the clinical syndrome of rhabdomyolysis [2]. It is important to note that multiple infectious agents, including HIV, can cause rhabdomyolysis [2]. High levels of HIV RNA in plasma and a sharp decline in CD4 cell count are hallmarks of acute HIV infection, which typically manifests two to six weeks after HIV exposure. Numerous vague symptoms are known to accompany acute HIV infection. However, presenting rhabdomyolysis as the first symptom is relatively uncommon [3]. By describing a case of acute HIV infection presenting as rhabdomyolysis, we underscore the need to consider HIV infection in the differential diagnosis of unexplained rhabdomyolysis.

## Case presentation

A 32-year-old male without a past medical history presented to the ED with complaints of fever, chills, decreased appetite, diffuse myalgias, generalized malaise, fatigue, and nausea. His social history was significant for having sexual intercourse with both male and female partners over the past few years, with intermittent condom use. He has no history of IV drug use. His initial physical examination was remarkable for a fever of 100°F, lymphadenopathy, and a diffuse maculopapular rash excluding palms and soles. He occasionally smokes cigarettes and marijuana and drinks one glass of wine daily. He denied any traumatic injury or recent travel.

His laboratory tests on admission revealed leukopenia, elevated alanine aminotransferase (ALT), aspartate aminotransferase (AST), and creatine phosphokinase (CPK) (Table 1). Urinalysis showed small amounts of blood on dipstick but no notable RBCs on microscopy. The HIV 1/2 Antigen/Antibody screen was positive. Confirmatory Western blot was negative for both HIV-1 and HIV-2 antibodies. However, HIV-1 RNA polymerase chain reaction (PCR) was detected at above 10,000,000 copies/mL, indicating acute HIV infection.

**Table 1:** Blood work on admission. ALT: Alanine aminotransferase; AST: Aspartate aminotransferase; Ab: Antibody; Ag: Antigen; Anti-HBcore: Hepatitis B Core Antibody; Anti-HCV Ab: Hepatitis C Antibody; CPK: Creatine phosphokinase; HBsAg: Hepatitis B Surface Antigen; HbsAb: Hepatitis B Surface Antibody; MCV: Mean Corpuscular Volume; PCR: Polymerase chain reaction; RNA: Ribonucleic Acid; RSV: Respiratory Syncytial Virus.

Variable	On admission	Reference range
WBC	3.9	4.2-9.1 10*3/uL
Hemoglobin	15.9	13.7-17.5 gm/dL
Hematocrit	46.3	40.1-51.0%
MCV	87.9	79.0-92.2 fL
Platelet Count	92	150-450 10*3/uL
ALT	264	4-36 IU/L
AST	976	8-33 IU/L
Alkaline phosphatase	50	38-126 IU/L
Bilirubin Total	0.2	0.1-1.2 mg/dL
CPK	20,129	38-174 IU/L
Calcium	8.2	9.2-11.0 mg/dL
Albumin	4.1	3.8-5.0 gm/dL
Creatinine	1.2	0.6-1.2 mg/dL
Urea Nitrogen	13	8-23 mg/dL
Sodium	136	135-145 mEq/L
Potassium	4.1	3.5-4.5 mEq/L
Lipase	54	22-51 U/L
HIV AG/AB	Reactive	Non-reactive
Confirmatory HIV 1/2 Ab	Non-reactive	Non-reactive
HIV RNA PCR	10,000,000	<20 HIV RNA/mL
Syphilis Screening	Non-reactive	Non-reactive
HBsAg	Non-reactive	Non-reactive
HBsAb	348.42	0.00-12.00 m[IU]/mL
Anti-HBcore	Non-reactive	Non-reactive
Anti- HCV	Non-reactive	Non-reactive
COVID-19	Non-reactive	Non-reactive
Influenza A and B	Non-reactive	Non-reactive
RSV	Non-reactive	Non-reactive
Alcohol	Non-detected	Non-detected

Following a consultation with the Infectious Disease service, serological tests for syphilis, hepatitis A, hepatitis B, hepatitis C, cytomegalovirus, Epstein-Barr virus, anti-nuclear antibodies, respiratory biofire, and a urine drug screen were performed, with all results being negative (Table 2). His CD4 count was 172/uL. Serum levels of alcohol, salicylate, and acetaminophen were undetectable. His abdominal ultrasound was reported as normal. 

**Table 2:** Blood work after admission. Ag: Antigen; Ab: Antibody; CD: Cluster of Differentiation; IgM: Immunoglobulin M; EBV: Epstein–Barr Virus; CMV: Cytomegalo Virus; ANA: Antinuclear Antibodies; IgG: Immunoglobulin G; LDH: Lactate Dehydrogenase; GGT: Gamma-Glutamyl Transferase.

Variable	After admission	Reference range
Biofire Respiratory	Not detected	Not detected
Salicylate	<4	10-20 mg/dL
Acetaminophen	<10	10-30 mg/dL
Absolute CD4 Helper cells	172	359-1519/uL
Absolute CD3	328	622-2402/uL
Absolute CD8 Supprosor cells	171	109-897/uL
Hepatitis A IgM Ab	Negative	Negative
Chlamydia	Negative	Negative
Gonorrhea	Negative	Negative
GGT	63	7-50 IU/L
LDH	750	140-280 IU/L
Ferritin	1113	15-200 ng/mL
EBV Ab IgM	<36	<36 U/mL
CMV Ab IgM	<30	<30 Au/mL
ANA	Negative	Negative
Urine Drug screen	Negative	Negative
Toxoplasma IgG	Negative	Negative
Toxoplasma IgM	Negative	Negative

**Figure 1:**
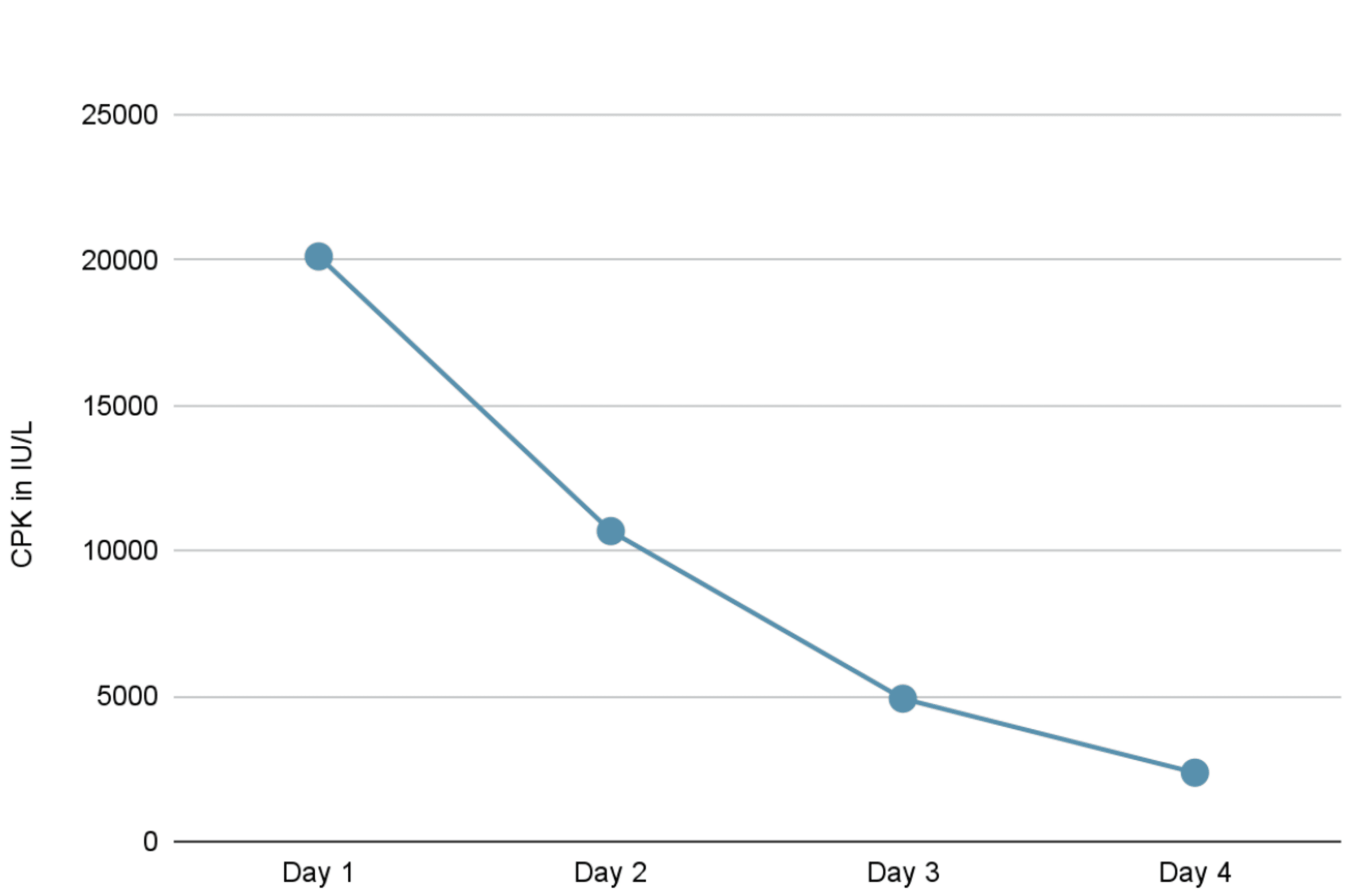
Creatine phosphokinase shows the decline in CPK levels after the patient was treated with IV fluids. CPK: Creatine phosphokinase.

Considering rhabdomyolysis secondary to acute HIV infection, the patient was started on IV fluids. His CPK downtrended to 2368 (Figure 1). Subsequently, his fatigue and rash improved. Genotypic resistance testing was conducted per infectious disease recommendations, revealing no evidence of antiretroviral resistance mutations. The patient was counseled, started on Biktarvy (bictegravir/emtricitabine/tenofovir), and discharged to follow up with the infectious disease clinic. His transaminases and CPK normalized during follow-up. His HIV-1 antibody seroconverted to positive after25 days. The patient remained adherent to antiretroviral therapy (ART), and his HIV RNA PCR decreased to 470 copies/mL at one month.

## Discussion

Rhabdomyolysis is characterized by the breakdown of muscle cells, releasing intracellular contents, including creatine phosphokinase, myoglobin, and electrolytes, into the bloodstream. It can be caused by various factors, including trauma, infection, drugs, and metabolic disorders [4]. Rhabdomyolysis is not uncommon in the HIV-positive population, particularly in those with advanced disease. A study reported that the prevalence of rhabdomyolysis is 9 per 1000 HIV patients. Risk factors unique to this population include HIV infection itself, opportunistic infections, co-infection with HCV, medication-related adverse effects (including pentamidine, trimethoprim-sulfamethoxazole, sulfadiazine, and antiretroviral agents such as zidovudine, raltegravir, and abacavir), drug-drug interactions, malignancy, and alcohol and/or illicit drug abuse [5, 6].

Acute HIV infection often presents with non-specific flu-like symptoms, such as fever, sore throat, or lymphadenopathy. Furthermore, unusual symptoms other than mononucleosis-like conditions can make diagnosing acute HIV infection more challenging [7, 8]. Acute HIV infection has been reported as an infrequent but potential cause of rhabdomyolysis. According to McDonagh CA and Holman RP, out of 23 patients reviewed, only four cases of rhabdomyolysis were more likely caused by acute HIV infection. The majority of case reports on rhabdomyolysis and acute HIV had certain limitations since inadequate investigation into alternative etiologies prevented HIV from being identified as the sole cause of rhabdomyolysis [9].

In this case, the patient's presentation with muscle pain led us to measure CPK levels. None of the classic causes of rhabdomyolysis were identified in our patient. Electrolytes were normal. Epstein-Barr Virus (EBV) and Cytomegalo virus (CMV), common viral infectious etiologies of rhabdomyolysis, tested negative. Toxic causes of rhabdomyolysis were ruled out as his urine drug screen and alcohol levels were normal. This clinical picture strongly indicated rhabdomyolysis induced by primary HIV infection, highlighting the importance of considering HIV infection in the differential diagnosis of rhabdomyolysis, especially in populations at risk for HIV. Rhabdomyolysis in acute HIV infection may be related to direct muscle invasion by the virus, immune-mediated mechanisms, or associated infections [10].

The treatment for rhabdomyolysis primarily involves early IV fluid resuscitation and elimination of the initial causative agent (such as discontinuing specific medications) [11]. Admission of patients with suspected rhabdomyolysis is essential for continuous intravenous hydration, diuresis, management of complications, and potential treatment of the underlying cause. Consider admission to an ICU in the presence of significant complications or metabolic disturbances [11]. When rhabdomyolysis is promptly and aggressively treated, the prognosis is generally favorable. Most cases of rhabdomyolysis that receive appropriate treatment are expected to achieve complete recovery of renal function, albeit requiring hospitalization. Regardless of the underlying cause of rhabdomyolysis, up to eight percent of cases may lead to mortality [12].

HIV treatment is recommended for acute HIV, and initiation of ART during acute infection may have several beneficial clinical outcomes, including improved preservation of immunologic function, significantly reduced time to viral suppression, and reduction of the viral reservoir [13]. In the context of initiating ART for HIV infection, the typical initial treatment regimen involves a combination of two nucleoside reverse transcriptase inhibitors along with a third active ART drug selected from one of three drug classes: an integrase strand transfer inhibitor, a non-nucleoside reverse transcriptase inhibitor, or a protease inhibitor [14].

## Conclusions

This case report illustrates the rare but clinically significant association between rhabdomyolysis and acute HIV infection. Clinicians should be vigilant in considering HIV infection as a potential cause of rhabdomyolysis, especially in patients at risk for HIV, even when typical HIV-related symptoms are absent. Early recognition and treatment of HIV infection are crucial to preventing further complications and improving patient outcomes.
